# ***T***umor-***I***nitiating ***C***ells: a cri***TIC***al review of isolation approaches and new challenges in targeting strategies

**DOI:** 10.1186/s12943-017-0602-2

**Published:** 2017-02-16

**Authors:** Komal Qureshi-Baig, Pit Ullmann, Serge Haan, Elisabeth Letellier

**Affiliations:** Life Sciences Research Unit, Molecular Disease Mechanisms Group, University of Luxembourg, 6, Avenue du Swing, L-4367, Campus Belval, Belvaux, Luxembourg

**Keywords:** Colorectal cancer, Cancer stem cells, Tumor-initiating cells, Culturing conditions, Surface markers, Spheroid Culture Systems, Metabolic identity, Targeted therapy, Inter- and intra-tumor heterogeneity

## Abstract

Most cancers contain a subpopulation of highly tumorigenic cells, known as cancer stem cells (CSCs) or tumor-initiating cells (TICs). Targeting TICs may be essential to achieve cure, because of their self-renewal and tumorigenic properties as well as their resistance to conventional therapies. Despite significant advances in TIC biology, their isolation and identification remain largely disputed and incompletely established. In this review, we discuss the latest developments in isolation and culturing approaches of TICs, with focus on colorectal cancer (CRC). We feature recent findings on TIC-relevant signaling pathways and the metabolic identity of TICs, as well as their current clinical implications. Lastly, we highlight the influence of inter- and intra-tumoral heterogeneity on TIC function and targeting approaches.

## Background

Colorectal cancer (CRC) is one of the most frequently diagnosed cancer types for both men and women and is the third most common cause of cancer mortality in Western countries [1]. Specific alterations in oncogenes and tumor suppressors are associated with the stepwise progression from normal colon mucosa to carcinoma, resulting in a growth and survival advantage of the affected cells. Notably, loss of function mutations in the adenomatous polyposis coli (*APC*) tumor suppressor gene on chromosome 5q21 are known to be among the earliest genetic events to take place in CRC [2]. In fact, loss of *APC* leads to a rapid Wnt deregulation and acquisition of a progenitor cell phenotype in the colonic crypt [3]. Although considerable advances have been made on the molecular mechanisms underlying CRC, it is still a matter of debate which mechanisms determine CRC initiation. The discovery of stem cells in colonic crypts supports the hypothesis that normal stem cells might accumulate tumorigenic mutations promoting malignant transformation, especially due to their long lifespan and their capacity to self-renew. In three break-through studies that represent a paradigm shift in cancer biology, cell lineage tracing within growing tumors revealed the presence of a tumor-driving subpopulation of cells in glioblastoma [4], squamous skin tumors [5] and intestinal adenomas [6]. In particular, Schepers and colleagues showed that cells positive for leucine-rich repeat-containing G-protein coupled receptor 5 (Lgr5) - a known Wnt target and marker for normal intestinal stem cells [7] - not only contribute to the initial stages of adenoma growth, but also display multipotent stem cell traits as they are able to generate all of the other cell types present in colon adenomas [6]. By tracing the contribution of individual cancer cells to tumor formation, these three studies provide direct experimental evidence supporting the hypothesis that some tumors contain a small population of cells displaying self-renewal and tumor initiation power, along a vast majority of cells that are non-tumorigenic. This subset of cells is referred to as tumor-initiating cells (TICs), also known as cancer stem cells (CSCs) or CSC-like cells, and can give rise to a heterogeneous population of cells similar in composition to the tumor of origin [8]. Many groups use the term "CSC" that reflects the existence of a cell at the apex of a differentiation hierarchy within tumors. We prefer to apply the term "TIC", which represents a functional definition as it refers to the capacity of these cancer cells to induce tumor formation in xenotransplantation studies. The clinical relevance of TICs is further corroborated by recent molecular classification studies, demonstrating that the expression of stem cell and mesenchymal genes denotes a CRC subtype associated with very poor prognosis [9–14].

Despite considerable advances in TIC biology, the isolation and identification of TICs still remain incompletely established. While some studies focus on an antigenic approach, others rely more on functional characteristics that define TICs. In this review, we will comment on the latest developments regarding isolation of colon TICs with focus on tumorspheres, also called spheroid culture (SC) models, derived from patients and cell lines. Additionally, we will discuss different culturing conditions (i.e. serum-containing conditions leading to adherent cultures and serum-deprived conditions favoring growth as spheroids, as well as medium switch experiments) in regard to the maintenance of TIC traits and possible TIC enrichment. Next, key signaling pathways and metabolic mechanisms that are involved in TIC regulation as well as their current clinical implications will be considered. Finally, we will highlight inter- and intra-tumor heterogeneity in cancer and especially in the TIC compartment and speculate how these new findings may impact the development of new TIC-targeting strategies.

## Isolation and identification of TICs

TICs were first described during the 1990’s in studies of leukemia stem cells [15, 16]. A few years later, TICs were also identified in solid tumors of different origins, among which breast [17], skin [18], brain [19, 20], pancreas [21], lung [22] and colon [23, 24]. Controversies still exist concerning the number of TICs within tumors. Indeed, the true frequency of TICs in most human tumors might be underrated due to obstacles encountered in the different techniques, one of the hurdles being the genetic background of the immune-deficient mouse strain used for xenotransplantation assays [25]. Nevertheless, TICs incline to be relatively infrequent in solid tumors [26, 27], although several exceptions exist, such as melanomas in which TICs reach up to 25% of the tumor population [25]. Within the TIC sub-compartment, the number of tumorigenic cells substantially varies between patients of the same tumor type [27]. Noteworthy, extensive stromal-niche interactions are critical for TIC survival and growth [28]. Thus, to accurately determine TIC frequency in human tumors, more "humanized" models replicating the tumor’s natural microenvironment - i.e. including a stromal and immune cell compartment - should be employed in the future [29].

TICs are defined by their self-renewal, differentiation and tumor-initiation capacities. They have been described to propagate tumors that are capable of recapitulating the heterogeneity of primary tumors [23, 24]. Different approaches are used to isolate TICs and while some of them are based on the expression pattern of cell surface markers, others rely more on the functional aspects of TICs. Most importantly, after isolation of the potential TIC-containing population, TICs are ultimately functionally identified based on their self-renewal potential, which is one of the main properties characterizing these cells. These assays have been extensively described [30, 31] and are not the focus of this review. Briefly, the self-renewal capacity is assessed in vitro by performing sphere formation assays under clonal conditions (including single-cell assays) and in vivo by transplantation experiments in mice. In the latter setting, limiting dilution assays (LDA) with serial tumor transplantation and subsequent tumor formation in secondary recipients are considered the gold standard in TIC research as they assess the most important biological trait of TICs, i.e. in vivo self-renewal.

### The antigenic approach

The antigenic approach takes advantage of a variety of cell surface markers, such as prominin-1 (commonly known as CD133), CD44, CD24, epithelial-specific antigen (EpCAM/ESA), CD166, CD29 and CD49f, or a combination of them (Tables 1 and 2) [7, 23, 24, 32–39]. Tables 1 and 2 summarize surface markers, either alone or in combination, used to identify colon TICs and illustrate their relevance based on the observed self-renewal capacity of TICs upon sorting for the marker of interest. Examples for markers that gave controversial results are CD133 and Lgr5 (Table 1). Over the last years, many reports have challenged the view of CD133 being a universal TIC marker [40–43]. Importantly, several in vivo studies show that CD133^+^ and CD133^−^ cells form tumors with similar efficiency [40, 43, 44]. Over the last years, Lgr5 has been revealed as a marker for normal and cancerous intestinal stem cells [7, 45]. Notwithstanding, the role of Lgr5 in CRC remains indistinct; while some studies suggest that intestinal tumors arise from Lgr5-positive cells [6, 45, 46], Walker and colleagues showed that suppression of Lgr5 expression enhances tumorigenesis [47].

**Table 1:** Colon TIC markers

			assessment of self-renewal		cellular system	
Marker	Known function	References	In vitro	In vivo	cell lines	patients
CD133	Regulation of cell membrane topology	[O’Brien et al., 2007] **1** [Ricci-Vitiani et al., 2007] **2** [Todaro et al., 2007] **3** [Vermeulen et al., 2008] **4**[Haraguchi et al., 2008] **5** [Ieta et al., 2008] **6** [Wang et al., 2012] **7** [Shmelkov et al., 2008] **8** [Dittfeld et al., 2009] **9** [Fan et al., 2014] **10** [Dubash et al., 2016] **11** [Qureshi-Baig et al., 2016] **12**	**1** ✓ **2** ✓ **3**✓ **4** ✓ **6** ✓ **7** ✓ **8 −** **9 −** **10 −** **11 −** **12 −**	**1** ✓ **2** ✓ **3** ✓ **4** ✓ **5** ✓ **6** ✓ **8 −** **9 −** **10 −** **11 −**	**6** **7** **9** **10**	**3** **1** **2** **4** **5** **8** **10** **11** **12**
LGR5	Cell adhesion, intestinal stem cell marker	[Kemper et al., 2012] **1** [Hirsch et al., 2014] **2** [Walker et al., 2011] **3**	**1** ✓ **2** ✓ * 3 – *	**1** ✓ **2** ✓	**1** **2** **3**	**1**
CD44	Cell adhesion and migration, cell-cell interactions, cell signaling, leukocyte attachment and rolling	[Dalerba et al., 2007] **1** [Vermeulen et al., 2008] **2** [Du et al., 2008] **3** [Haraguchi et al., 2008] **4** [Chu et al., 2009] **5** [Yeung et al., 2010] **6** [Chen et al., 2011] **7** [Wang et al., 2012] **8** [Ohata et al., 2012] **9**	**2**✓ **3**✓ **5**✓ **6**✓ **7**✓ **8**✓ **9**✓	**1**✓ **3**✓ **4**✓ **5**✓ **6**✓ **7**✓ **9**✓	**7** **8**	**1** **2** **3** **4** **5** **6** **9**
CD44v6	CD44 variant isoform, cell migration and invasion	[Todaro et al., 2014] **1**	**1**✓			**1**
CD24	B cell proliferation and maturation	[Vermeulen et al., 2008] **1** [Yeung et al., 2010] **2** [Ke et al., 2012] **3**	**1**✓ **2**✓ **3**✓	**2**✓ **3**✓	**2** **3**	**1**
CD166	Cell adhesion and cell-cell interactions	[Dalerba et al., 2007] **1**		**1**✓		**1**
EpCAM	Cell adhesion, migration, signaling	[Dalerba et al., 2007] **1**		**1**✓		**1**
EphB2	Position of the different cell types in the crypts	[Merlos-Suárez et al., 2011] **1**		**1**✓		**1**

✓: correlation between self-renewal capacity and expression of surface marker

−: no correlation between self-renewal capacity and expression of surface marker

*: studies based on gene silencing

**Table 2:** Colon TIC marker combinations

		assessment of self-renewal		cellular system		
Marker combinations	References	In vitro	In vivo	cell lines	patients	mouse
CD166^+^/CD44^+^	[Dalerba et al., 2007] **1**		**1**✓		**1**	
EpCAM^high^/CD44^+^	[Dalerba et al., 2007] **1** [Kai et al., 2009] **2**	**2**✓	**1**✓ **2**✓	**2**	**1**	
CD24^high^/CD29^+^	[Ghazvini et al., 2013] **1**		**1**✓			**1**
CD133^+^/CD44^+^	[Haraguchi et al., 2008] **1** [Chen et al., 2011] **2**	**1**✓	**1**✓ **2**✓	**1** **2**	**1**	
CD133+/CD49f+	[Haraguchi et al., 2013] **1**		**1**✓		**1**	
CD44+/CD49f+						
CD24^+^/CD44^+^	[Yeung et al., 2010] **1**	**1**✓	**1**✓	**1**		
CD44^+^/CD133^−^	[Wang et al., 2012] **1**	**1**✓		**1**		
CD133^+^/CD24^+^	[Vermeulen et al., 2008] **1**	**1**✓			**1**	
CD133+/CD24-	[Vermeulen et al., 2008] **1** [Haraguchi et al., 2008] **2**	**1** − **2** −	**2** −	**2**	**1** **2**	
CD133+/CD44-						
CD133+/CD44+						
CD133+/CD166-						
CD133+/CD166+						
CD166+/CD44+	[Muraro et al., 2012] **1**	**1** −	**1 **−	**1**		
CD24+/CD44+						
CD44+/CD166+/EpCAMlow	[Collura et al., 2013] **1**	**1** −		**1**		
CD44+/CD166+/EpCAMhigh						
CD133+/CD26+/CD44+ CD133+/CD26+/CD44-	[Pang et al., 2010] **1**	**1**✓	**1**✓		**1**	
CD133+/CD26-/CD44+						
CD133+/CD26-/CD44-						
CD133-/CD26+/CD44+ CD133-/CD26+/CD44-						
CD133-/CD26-/CD44+						
CD133-/CD26-/CD44-		**1** −	**1** −		**1**	

✓: correlation between self-renewal and expression of surface markers

−: no correlation between self-renewal and expression of surface markers

Notably, culturing conditions including cell density and passage number as well as extrinsic factors are suggested to largely influence surface marker expression ([48–51] and cf. next paragraph). Furthermore, there is a large inter-patient variability in the expression of surface markers, with no or small expression to high positivity for the same marker across patients [41, 42, 52–54]. This limited overlap between the phenotype of TICs isolated from different patients of the same tumor type most probably reflects the presence of heterogeneous and biologically distinct TIC pools, which might render the identification of TICs difficult and biased. Additionally, as TICs represent a highly dynamic population, it will be important to better understand the influence of the microenvironment on the antigenic profile of TICs. This inter- and intra-tumor heterogeneity as well as its impact on TIC marker expression will further be discussed in the last paragraph of this review.

Several cell surface markers including CD44, CD166 and EpCAM, are players in cell adhesion and attachment, and thus have been thought to favor the survival of tumor cells within the microenvironment [32]. As these markers are not exclusively expressed by TICs [55], but also by other cells, among which stromal cells, their use to isolate TICs from tumor tissue is precarious. Very recently, CD166 was shown to be expressed in stromal progenitor cells within the hematopoietic niche [56]. Furthermore, CD44, which is described to mark colon TICs, includes multiple splice variants. Original TIC isolations were performed by using pan-CD44 antibodies [32], but recently it was shown that full length CD44 is more widely expressed, and that TICs are better identified with the CD44v6 splice variant [57]. Overall, the use of surface markers in the aim to identify and isolate colon TICs remains delicate.

### The functional approaches

#### Label-retaining methods

There are several approaches to identify TICs based on their functional characteristics. TICs are thought to be relatively quiescent, displaying slow proliferative properties, and giving rise to two daughter cells by asymmetric cell division [58]. Thus, TICs can be isolated based on their quiescent traits through the use of lipophilic dyes, such as PKH26 or PKH6. While a cell undergoing a slow division effectively retains the dye, a fast dividing cell rapidly loses or dilutes it from the membrane. TICs retain the dyes for longer periods than the differentiated daughter cells [59, 60]. Bromodeoxyuridine (BrdU) labeling is based on a similar label retention approach. TICs retain more BrdU compared to differentiated cells as it dilutes in dividing cells [39].

#### Side-population assay

Another functional trait of TICs is that they display a lower Hoechst dye staining pattern. It is known that an increased expression of membrane proteins of the ATP-binding cassette (ABC) family, which pump various small molecules (such as cytotoxic drugs and dyes) out of cells, is in part responsible for the dye efflux [61]. The resulting fraction displaying a lower Hoechst gradient is called side population (SP). Whereas some studies speculate that SP cells in CRC contain an increased TIC fraction [62, 63], others could not associate SP cells with enriched TIC properties, such as clonogenic and multipotent differentiation potential [64]. Over decades, the use of SP assays to identify TICs has come along with questionable interpretations (reviewed in [65]). The SP phenotype is not exclusive to stem cells and has also been described in various differentiated cells in adult tissue [66–68]. Thus, caution is required while applying this assay for the identification of TICs. Especially, tumor and stromal cell compartments need to be discriminated and diploid versus aneuploid cell populations should be considered for the analysis [65].

#### Isolation of TICs based on autofluorescence

Miranda-Lorenzo and colleagues recently presented a novel strategy for the isolation and identification of TICs across different human tumor types, including CRC [53]. Their approach was based on cells with an autofluorescent subcellular compartment that displayed essential TIC-specific properties, such as self-renewal, long term tumorigenicity and invasiveness in vivo. The distinct autofluorescent population of self-renewing and highly tumorigenic TICs harbored an inherent ability to concentrate the fluorescent vitamin riboflavin in intracellular vesicles that were coated with ATP binding cassette subfamily G member 2 (ABCG2), an ATP-dependent transporter. Even if this small subset of autofluorescent cells display TIC properties, a functional role for the accumulation of riboflavin in autofluorescent vesicles could not be established in regard to TIC biology [53]. This assay might represent a new approach to identify TICs; however, it needs to be validated by other TIC studies. Noteworthy, stromal cells such as macrophages display high autofluorescence [69], which may limit the use of this assay.

#### Alternative approaches based on the metabolic identity of TICs

An emerging strategy to identify TICs is based on metabolic and bioenergetic differences between TICs and their non-tumorigenic counterparts. Indeed, emerging evidence indicates that cellular metabolism and stemness are strongly intertwined processes [70]. Embryonic and adult stem cells have a reduced number of mitochondria and display a decreased oxygen consumption rate, thereby displaying a rather glycolytic than oxidative metabolite and gene expression signature [71, 72]. During differentiation, stem cells undergo a "metabolic shift" from active glycolysis to enhanced aerobic mitochondrial respiration [73]. As TICs and stem cells are known to share common properties (i.e. their self-renewal and differentiation abilities), it seems reasonable to assume that TICs are also subject to metabolic reprogramming. Although pancreatic [74] and glioma [75] TICs were found to mainly rely on mitochondrial respiration, many studies on other cancer types, including osteosarcoma [76], melanoma [77], as well as lung [78], breast [79], and liver [80] cancer agree that TICs preferentially display a glycolytic phenotype and reduced mitochondrial activity. Opposing results were found for CRC and hence the metabolic status of colon TICs is still under debate [81]. While Song and colleagues state that high activity of mitochondrial metabolism is required for growth of colon TICs [82], Schell et al. stress that TICs actively suppress oxidative phosphorylation by inhibiting pyruvate import [83].

These conflicting findings may arise from differences in TIC isolation and cultivation techniques; microenvironmental stimuli, such as nutrient starvation, oxidative stress or hypoxia, influence the metabolic state of TICs [84, 85]. Tumor hypoxia has been shown to further potentiate the glycolytic phenotype of TICs. Besides inducing the expression of glycolytic genes, hypoxia-inducible factor 1α (HIF-1α), is known to actively suppress mitochondrial respiration by promoting pyruvate dehydrogenase kinase 1 (PDK1), ultimately resulting in repressed pyruvate dehydrogenase (PDH) and reduced TCA cycle activity [86]. Along similar lines, we recently showed that hypoxic culture conditions result in microRNA-210-induced metabolic reprogramming of colon TICs from mitochondrial respiration to increased lactate production. This glycolytic phenotype correlated with enhanced tumorigenicity and self-renewal capacity of colon TICs [85]. Taken together, controversy remains concerning the precise bioenergetic identity of TICs. Thus, a better metabolic characterization of tumorigenic and non-tumorigenic cancer cells may lead to more reliable TIC-specific identification methods in the future.

Another approach relies on the difference in the aldehyde metabolism that exists between TIC and cancer cells. Aldehyde dehydrogenases (ALDHs) are a family of cytosolic isoenzymes that are responsible for oxidizing intracellular aldehydes, leading to the oxidation of retinol to retinoic acid and protecting the organism from damage induced by active aldehydes [87]. In particular the assessment of aldehyde dehydrogenase 1 (ALDH1) activity has been widely used to identify TICs in various cancer types [87]. Although ALDH1 activity assays show controversial results in the context of pancreatic TICs [53], ALDH1^+^ CRC cells are reported to display increased TIC traits, especially increased self-renewal capacity and tumorigenicity, compared to the ALDH1^−^ fraction [36, 41].

#### Spheroid culture systems

TICs are able to self-renew and display anchorage-independent growth in form of spheroids, a trait that can be used to enrich for TICs in various cancer types [18, 19, 21, 24, 42, 52, 88–91]. Most often, 3D in vitro SC systems that use low-adherent conditions include the use of serum-free medium supplemented with specific growth factors to allow for TIC enrichment. These models are often referred to as tumorspheres or spheroid culture (SC) systems, in which differentiated and non-malignant cells undergo anoikis due to the lack of adherence [92]. Importantly, SC conditions allow to efficiently eliminate non-malignant cell types, such as fibroblasts, that are present in the freshly resected primary tumor tissue and that may outcompete and eventually outgrow cancer cells under serum-containing conditions [23, 24, 42, 43]. This assay has however some disadvantages as it imposes the use of specific culture conditions with consequences on the cancer cell phenotype. Indeed, it is not clear whether the applied conditions select for TICs that originally exist or merely drive cancer cells to adapt a cancer stem cell phenotype. In addition, controversies still exist on whether SCs comprise a homogenous population enriched in undifferentiated cells [89, 93] or rather a large range of morphologically different entities, which show inter- and intra-sphere molecular heterogeneity, including variable expression of markers [91, 94]. These questions might soon be addressed by applying modern technologies such as imaging flow cytometry combined with single cell sequencing. Albeit some exceptions exist [95], most studies report that cells derived from SCs display a high self-renewal capacity in vitro, which correlates with a pronounced tumor-initiating capacity upon injection of low cell doses into immune-depressed mice [24, 42, 96]. Additionally, long-term passaging of cells under spheroid culture conditions further allows for the enrichment in colon TICs over time [42, 91]. Furthermore, SCs have been shown to faithfully preserve key characteristics of the original patient tumors, including gene expression profiles, tumor heterogeneity and tumor morphology, as well as relevant mutations [24, 42, 54, 88, 90, 91].

#### Chemoresistance

TICs have been described to display extensive chemoresistance characteristics. In fact, TICs are able to evade DNA damage by reducing the production of ROS and by enhancing the activity of DNA checkpoint kinases [97, 98]. Furthermore, TICs appear to express high levels of ATP-binding cassette (ABC) transporters, potentially excreting antitumor drugs, and thus contributing to treatment resistance [99–102]. These latter properties represent the underlying principle of the SP assay that has been described earlier. Additionally, functional chemoresistance capacity should be considered as a supplementary feature displayed by TICs, rather than a stringent functional property and thus might not be used as a method for TIC isolation.

#### Organoids

Over the last years, intestinal epithelial organoid cultures have emerged as a new system to expand and study intestinal crypts [103]. Organoids allow intestinal stem cells to maintain both their self-renewal capacity and differentiation hierarchy, similar to how it is observed in the adult intestine in vivo. The use of patient-derived organoids from CRC tumors and tumor-associated normal tissue, constituting an ideal matched control, further enables the testing of a range of therapeutic compounds in a patient-relevant model. Finally, long-term organoid cultures of primary CRC cells might prove a suitable system to study colon TIC biology in a more physiologically relevant setting and thus their use may lead to advancement in CRC treatment.

## The influence of different culture conditions on TIC traits

It is still not clear to which extent different culture conditions (i.e. serum-deprived conditions favoring growth as spheroids and serum-containing conditions leading to adherent cultures) influence TIC features. While some studies in CRC could show that self-renewal capacity is increased in SCs compared to adherent counterpart cultures [24, 90], others did observe similar functional properties of TICs between both culturing conditions [41, 104–107]. Calvet and colleagues, suggest that SCs enrich for TICs in a cell line-dependent manner [106]. Colon spheres derived from the Caco-2 cell line lose several TIC properties compared to their parental adherent counterpart [107]. Similarly, SCs derived from the CRC cell line HCT116, were described to follow a more stochastic than hierarchal organization [108]. These conflicting observations may be explained by the dynamic regulation of TIC properties. Noteworthy, TIC features, including marker expression, are largely influenced by extrinsic factors such as culturing conditions [48–51]. It may be assumed that the loss of expression of a given marker does not alter the tumorigenic potential of TICs. Alternatively, dedifferentiation events could induce the formation of a specific TIC subpopulation with an antigenic profile that is similar to non-tumorigenic cells whereas the functional phenotype is retained [109]. In yet another scenario, acquired mutations and clonal evolution of TICs might lead to the generation of specific sub-clones. These subpopulations may show reduced tumorigenic potential while maintaining TIC-like surface marker expression. Modern technologies, such as single cell sequencing or cell lineage tracing, are currently being used to further investigate these issues.

**Fig. 1:**
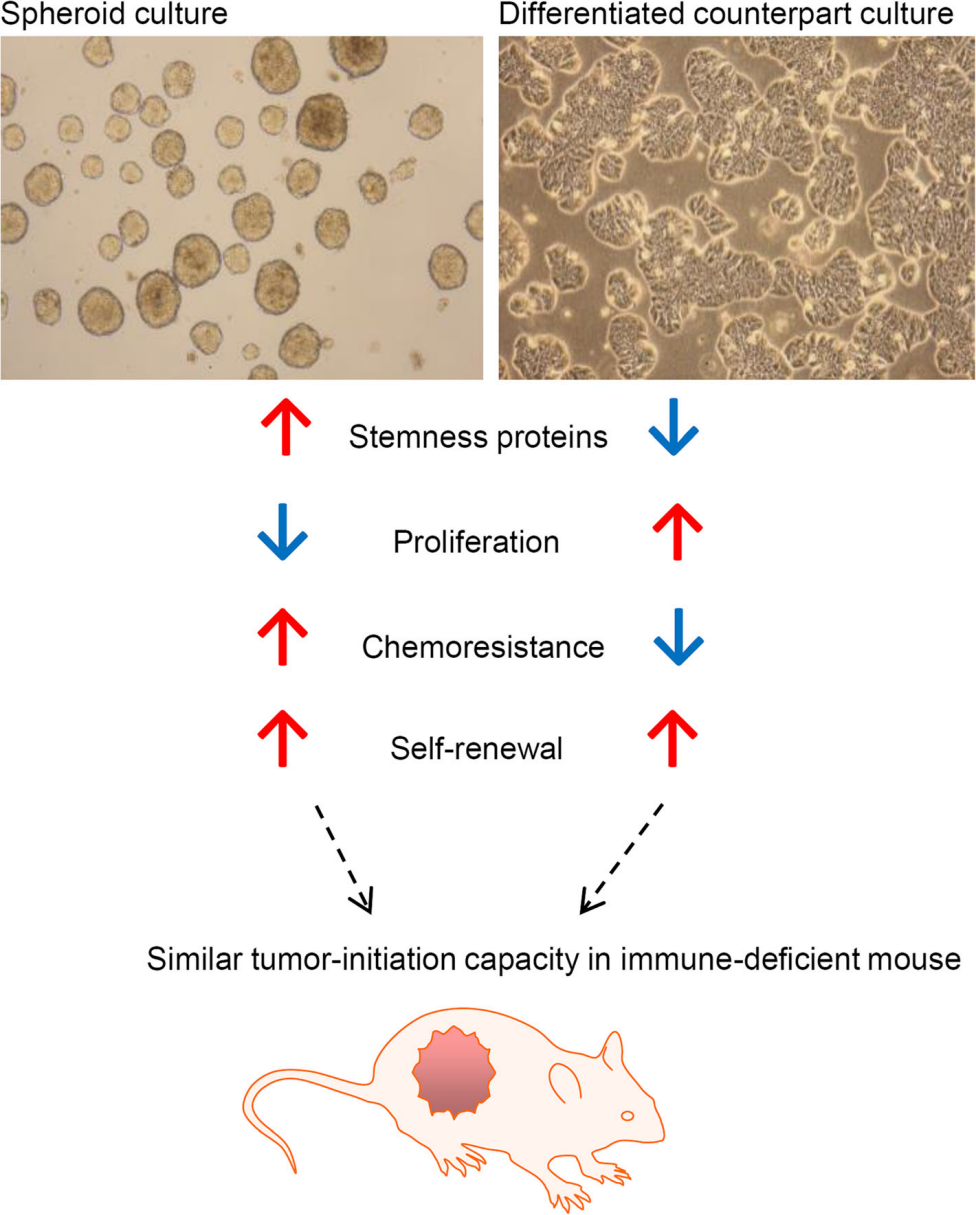
TICs display pronounced plasticity:﻿ self-renewal as well as tumor-initiation capacities of TICs are not restricted to phenotypically immature cells. Spheroid cultures display increased chemoresistance and expression of stemness markers, as well as reduced proliferation, compared to adherent differentiated counterparts. However, both spheroids and adherent counterparts have comparable self-renewal capacities and can lead to similar tumor formation when low cell numbers (10 cells per injection) are injected subcutaneously into immune-deficient mice

To further interrogate the influence of different culturing conditions on TIC features, we have compared cells derived from traditional CRC cell lines or tumor biopsies, cultured either as SCs (i.e. serum-deprived culture conditions with growth factors) or as regular adherent cultures (i.e. serum-containing culture conditions), respectively [42]. In comparison to adherent counterpart cultures, SC-derived cells display a decreased expression of the differentiation marker CK20 and an increased expression of stemness proteins, such as sex determining region Y-box 2 (Sox2), octamer-binding transcription factor 4 (Oct4), Nanog as well as Lgr5, a property which is common to both stem cells and their tumorigenic counterparts [110]. By seeding single cells per well and monitoring sphere formation over time, we demonstrated that SCs derived from traditional CRC cell lines and tumor tissue show high self-renewal capacity. Nevertheless, even after long-term culture in TIC-enriching conditions, SCs that are transferred to differentiating culture conditions (i.e. serum-containing conditions) still have the capacity to adhere and morphologically resemble differentiated cell populations or the parental cell lines. Likewise, when long-term SC-derived adherent differentiated cultures are reversed to TIC conditions (i.e. serum-deprived conditions), they are able to form spheres to a similar extent as the initial SCs [42]. It could further be observed that spheroids and the spheroid-derived adherent differentiated cultures display similar self-renewal capacity and equally form tumors in immune-deficient mice [42, 43]. Additionally, clonal analysis of individual lentivirally marked clones in spheroid cultures and adherent counterparts revealed no systematic differences in contributing clone numbers [43]. These findings suggest that self-renewal and tumor-initiation capacity of TICs might not be restricted to phenotypically immature spheroid cells, and furthermore underlines the high plasticity of cancer cells that are able to reacquire stem-cell traits even after long differentiation processes, a feature that needs to be closely examined for the development of TIC-specific therapies [111] (Fig. 1).

In a study by Collura and colleagues, an extensive characterization of 25 established CRC cell lines was performed and it was shown that SCs do not seem to present enhanced TIC traits in regard to tumor-initiating potential but display chemoresistance ability, compared to adherent cultures [101]. Similarly, we demonstrated that primary SC-derived cells display chemoresistance to 5-fluorouracil (5-FU), compared to adherent differentiated cells in different experimental settings [42]. It would now be important to extend these data to an in vivo setting. Interestingly, SCs from established cell lines were more sensitive to chemotherapy than primary SCs derived from patients, highlighting that primary tumorspheres maintain pronounced chemoresistance and thus more closely reflect patient response [42]. It may be speculated that certain observed TIC features, such as higher chemoresistance, are due to phenotypic differences that occur in the TIC compartment over long periods of cell culture. In this context, we have reported that original patient tumor material and primary established cultures share a similar mutational profile, which is also present in the respective adherent counterparts, excluding the possibility that the observed resistance of TICs to chemotherapeutics is due to differences in mutations that have arisen over time or after application of different culture conditions [42]. We might further hypothesize that primary patient-derived SCs harbor increased expression of ABC transporters or Lgr5, compared to adherent cultures or to cell-line derived SCs. Alternatively, intra-tumoral heterogeneity might be lost in cell-line derived TIC cultures whereas primary established cultures, used at early passages, could better retain this clinically relevant feature.

Besides differing in means of chemoresistance, SCs further differ from their adherent counterparts in terms of proliferation rate. We observed that spheres derived from differentiated cultures were bigger in size (correlating with a more proliferative phenotype), compared to spheres from SCs. This observation might emphasize the slow-proliferative and potentially stem-like properties of TICs specifically in SCs compared to the adherent counterparts [42], possibly providing SCs with a chemoresistance advantage. Taking the new findings into consideration, SCs seem to represent a superior model to adherent differentiated counterparts for screening of new CRC therapies. The superiority of the SC model is mainly due to the resistance to chemotherapeutics, which is especially retained in primary tumor-derived SCs and which more closely reflects the therapeutic response observed in patients.

## TIC-targeting approaches and clinical implications

The tumor-initiating and chemoresistant features of TICs highly encourage the development of specific TIC-targeting treatments. Conventional cancer therapies do not discriminate between TICs and rapidly growing cancer cells. Whereas temporary regression of the tumor mass might be achieved through targeting differentiated cancer cells, TICs can remain mostly unharmed. New tumors may arise through the tumor-promoting effects of TICs, thereby leading to a rapid relapse of the malignancy. TIC-specific antitumor treatments might be unable to induce rapid shrinkage of the tumor bulk, but instead may eliminate the capacity of TICs for long-term growth, ultimately leading to tumor growth arrest [112].

The identification of differences in metabolic regulation between differentiated cancer cells and TICs has led to the development of several new TIC-specific treatment strategies, such as oxidative stress-based therapies, nitric oxide synthase inhibition, or blockade of aerobic glycolysis [113]. TIC self-renewal is known to be dependent on low levels of ROS [114]. The detoxifying enzyme ALDH1, which is often considered to be a reliable TIC marker, was shown to protect colon TICs against excessive oxidative stress [36]. Accordingly, pharmacological repression of ALDH might kill TICs by selectively inducing ROS production in these cells. Consistently, Chiba et al. could show that the application of disulfiram, a selective ALDH inhibitor, resulted in diminished self-renewal activity and a reduced number of liver TICs [115]. Besides, TICs were shown to display increased nitric oxide (NO) synthesis levels [116] and tumorigenic capacity; and growth of colon TICs could be reduced by using specific inhibitors of inducible nitric oxide synthase (iNOS) [117].

Another promising approach to specifically eradicate TICs might be to target their glycolytic phenotype. The anti-diabetic drug metformin has recently been tested in the context of CRC [118] and strong experimental evidence suggests that metformin, due to its interference with glucose homeostasis, selectively eliminates osteosarcoma [119], glioblastoma [120], and breast [121] TICs. Along the same line, we observed that hypoxia promotes the self-renewal capacity of colon TICs by activating microRNA-210 and by repressing TCA cycle activity [98]. Interestingly, high lactate levels originating from enhanced glycolysis are known to exert several pro-tumorigenic functions. Besides generating an acidic microenvironment, which is commonly associated with increased metastasis formation [122], hypoxia-induced lactate is known to reduce the activity of pH-sensitive T cells, thereby contributing to the immune evasion of tumor cells [123]. Moreover, high lactate levels are thought to generate TICs with a stem cell-like gene expression profile [80]. Importantly, we and others have shown that targeting lactate production efficiently represses the tumorigenic potential of TICs [78, 85], further strengthening the position of lactate as an important oncometabolite and highlighting the therapeutic relevance of glucose metabolism.

TICs display many features of embryonic or tissue stem cells, and preferentially demonstrate persistent activation of one or more highly conserved signal transduction pathways involved in development and tissue homeostasis [124–126] (Table 3). By aiming at the regulation of TIC maintenance and self-renewal processes, it might be possible to target this rare subpopulation [8, 127]. Accordingly, pathways such as Wnt/β-catenin, Notch, TGF-β, JAK/STAT and Hedgehog, which govern TIC growth and survival, are being addressed for therapeutic purposes [124–127] (Table 4). Wnt ligands that are produced from cells in the stem cell microenvironment serve as a self-renewal signal for normal stem cells and their tumorigenic counterparts and might therefore be interesting candidates to target TIC-relevant mechanisms [126, 127]. For instance, OMP-18R5, a monoclonal antibody currently in clinical trial phase I, was shown to impair the self-renewal capacity of TICs by targeting the Wnt receptor FZD7 and to inhibit the growth of breast, pancreatic, and colon cancer [128]. Multiple trials involving Wnt/β-catenin inhibitors combined with current therapies are in progress (https://clinicaltrials.gov).

**Table 3:** Signaling pathways implicated in TIC regulation

Signaling Pathways	Property	References
Wnt/β-catenin	Self-renewal	[111, 126, 127, 152–155]
BMI-1	Self-renewal, stemness	[135, 156–158]
ID1/ID3	Self-renewal	[96, 159]
Hedgehog	Self-renewal, stemness	[127, 134, 160–162]
Notch	Self-renewal	[127, 131, 132, 163–165]
JAK/STAT	Self-renewal, stemness, tumorigenic potential	[138–140, 166–171]
TGF-β	EMT, stemness, dual role in CRC	[10, 125, 172]

**Table 4:** TIC-targeting drugs under clinical investigations. Adapted from [81, 125, 173–175]

Target molecules	Therapeutics	Disease	Clinical trial	Company
Undisclosed	TIC inhibitor BB1608	CRC	Entering phase III	Boston Biomedicals, Inc
Telomerase inhibitor	IMETELSTAT	Broad range	Phase II	Geron Corporation
CD133	Dendritic cell-based vaccine ICT-121	Glioblastoma	Entering phase I	ImmunoCelllular Therapeutics Ltd
Focal adhesion kinase inhibitor	VS6063	Advanced solid tumors	Phase I completed	Verastem and Pfeizer
Wilms Tumor 1	Peptides from Wilms Tumor 1 (FPI-01)	Leukemia and mesothelioma	Phase II	Formula Pharmaceuticals
EphA3	Human monoclonal antibody (KB004) binds EphA3	Leukemia	Phase I	KaloBios Pharmaceuticals, Inc.
Notch pathway	Anti-DLL4 (demcizumab) (OMP-21 M18)	Solid tumors	Phase II	OncoMed
	Anti-Notch2/3 (OPM-59R5)	Solid tumors	Phase I	
Wnt pathway	Anti-Fzd7 (OMP-18R5, vantictumab, binds 5 Frizzled receptors)	Solid tumors	Phase I	
	Truncated Frizzled 8-Fc fusion protein (OMP-54 F28)	Advanced solid tumors	Phase I	
Undisclosed cancer stem cell antigen	Peptides vaccine (SL401 and SL701)	Advanced leukemia and advanced brain cancer	Phase I/II completed	Stemline Therapeutics

An alternative approach to target TICs is to induce their differentiation. For this, bone morphogenetic protein 4 (BMP4) has been described to induce differentiation and to stimulate apoptosis in colon TICs. BMP4 acts by reducing β-catenin activation through inhibition of the PI3K/AKT pathway and activates Wnt-negative regulators [129, 130]. Similarly, delta-like canonical Notch ligand 4 (DLL4), which is an important component of the Notch pathway, contributes to stem cell self-renewal and vascular development. Notch pathway blockade through an anti-DLL4 antibody, which is in clinical trial phase II, has been shown to abolish relapse after chemotherapy in vivo [131, 132].

Another TIC-specific strategy is addressing survival pathways of colon TICs by inhibiting the interleukin 4 (IL-4) signal transduction pathway with an anti-IL-4 neutralizing antibody or an IL-4 receptor alpha antagonist to sensitize TICs to 5-FU and oxaliplatin. This effect was mainly achieved through a down-regulation of anti-apoptotic proteins, like cFLIP, BCL-xL and PED [90, 133].

The hedgehog pathway plays a role in maintaining stemness and self-renewal of TICs via the B lymphoma Mo-MLV insertion region 1 homolog, polycomb ring finger (BMI-1) [134], which is known to regulate the self-renewal of TICs in CRC [135]. BMI-1 forms an essential component of the polycomb regulatory complex 1 (PRC1). PRC1 has an important role in the organization of chromatin structure, which, in turn, regulates the expression of genes involved in stem cell behavior [136]. Inhibition of the hedgehog signaling pathway decreases TIC stemness via BMI-1 downregulation and, at the same time, reduces TIC chemoresistance via downregulation of ABCG2 [134]. Furthermore, treatment of primary CRC xenografts with a BMI-1 inhibitor was shown to result in the loss of colon TICs with long-term and irreversible impairment of tumor growth in mice [135].

Along the same lines, inhibitor of DNA binding (ID) 1 and 3 were shown to function together to influence the self-renewal of colon TICs through cell-cycle restriction driven by the cell-cycle inhibitor p21 [96]. Regulation of p21 by ID1 and ID3 was presented as a central mechanism preventing the accumulation of excess DNA damage and subsequent functional exhaustion of TICs in CRC. Furthermore, abolishment of ID1 and ID3 increased sensitivity of these cells to chemotherapy [96].

The Signal Transducer and Activator of Transcription 3 (STAT3), a mediator activated by members of the janus kinase (JAK) family, is known to play a role in the regulation of TICs. STAT3 cooperates together with NANOG and OCT4 and initiates transcription of stemness genes required for modulating pluripotency [137]. The STAT3 signaling pathway is implicated in the clonogenic and tumorigenic potential of prostate [138], colon [139] and breast TICs [140]. ALDH^+^ and CD133^+^ colon TICs exhibit a higher level of STAT3 phosphorylation compared to ALDH^−^, CD133^−^ or unsorted cells [139] and targeting the STAT3 signaling pathway was recently shown to reduce ALDH^+^ breast TICs [140]. In addition, blockade of STAT3 activity leads to the inhibition of tumor growth and tumor-initiating potential in CRC [139].

The transforming growth factor-β (TGF-β) signaling pathway is one of the most commonly altered pathways in human cancers. This pathway regulates cell proliferation, differentiation, migration, apoptosis and reportedly stem cell maintenance and function [141]. It is important to mention that TGF-β has a dual role and can switch from being a tumor suppressor to a tumor promoter, depending on the cell type and microenvironmental signals [141]. Thus, targeting TGF-β signaling for clinical development should be done with caution. Besides, aiming at targeting the immune cell response has emerged as a potential strategy to target TICs in various cancer types. This approach has been used in the context of CRC [142], acute myeloid leukemia (AML) [143] and human bladder cancer [144]. The resulting blockade of the immunoglobulin-like CD47 protein rendered the subpopulation of TICs susceptible to innate and adaptive immune system clearance by restoring phagocytosis by macrophages [143, 144].

## Inter- and intra-tumor heterogeneity: future challenges for TIC-specific treatments

Recent large-scale sequencing studies have revealed different molecular subtypes of CRC [9–14], demonstrating that it is not a uniform disease but a plethora of disparate tumor types and subtypes. This inter-tumoral heterogeneity, consisting of differences between individual patients, presents a significant hurdle to the eradication of cancer and led to the implementation of personalised medicine in the clinics. Besides this interpatient variability, intra-tumor heterogeneity denotes the coexistence of different populations of tumor cells that diverge in their genetic, phenotypic or behavioral characteristics within a given primary tumor [145]. Genetic, epigenetic as well as microenvironmental cues, which favor the growth of some cancer cells and the attrition of others, are thought to be the origin of such intra-tumor heterogeneity [145, 146]. Furthermore, spatial and temporal heterogeneity are common attributes in CRC and other tumor types. Thus, biopsies of small tumor pieces may not reflect the wide range of alterations found in the tumor as a whole. Altogether, inter- and intra-tumor heterogeneity is thought to largely contribute to therapy failure and disease progression [145]. Latest molecular biology tools, such as barcode sequencing, single cell analysis, lineage tracing, or whole-genome sequencing might help to face the challenge of dissecting both inter- and intratumor heterogeneity.

Recent studies have illustrated that clonal evolution is also occurring within the TIC population itself, with tremendous regulatory impact on self-renewal and tumor-initiation potential [147]. Indeed, mutational analysis of lymphoid leukemia cells demonstrated that individual tumors contain subclones that are genetically different but evolutionarily related [148]. Accordingly, therapeutic targeting of TICs turns out to be more challenging than was initially anticipated, as TICs are not static and genetically homogeneous entities. Along this line, Dieter and colleagues have shown the existence of three different types of TICs in primary human CRC, among which a rare subset of cells that maintain tumor growth in serial transplantation, one subset with limited self-renewal capacity and finally a more latent subtype that is only present in secondary recipients [52]. Additionally, by using lentiviral lineage tracing in combination with in vivo serial transplantation experiments, Kreso and colleagues could detect functional diversity among colon TICs that were derived from the same subclone: while some cells displayed long-term self-renewal potential and were detected in every transplant, others were less persistent, losing their proliferative capacity over time [149]. Most intriguing was probably the existence of a dormant subclonal species that became dominant following chemotherapy [149]. It will now be important to link these different functional phenotypes to a genomic and transcriptomic profile. High throughput sequencing platforms will for instance allow clinicians to better understand patient tumors and thus elaborate improved treatment approaches that aim exploiting subclonal-specific alterations. In this regard the organoid technology is of great interest, as recent studies have shown that organoids 1) recapitulate the properties of the original tumor 2) are amenable to high-throughput drug screening and most importantly 3) allow for the implementation of personalized medicine [103]. Indeed, multiple organoid cultures can be established from single cells of individual tumor clonal lineages present in the primary tumor [150]. Outgrowing clones may thereby be identified by applying sequencing approaches and treated in vitro with both standard cancer therapies and therapies tailored to the specific genetic program [147]. In the future, this strategy may demonstrate whether individual clones are sensitive to given therapies and could allow clinicians to decide for appropriate follow-up treatment regimens. Such approaches might help to identify and successfully eradicate the totality of pertinent tumor clones, ultimately preventing disease progression and relapse.

Additionally, inter- and intra-tumor heterogeneity arises from the high plasticity of TICs. Indeed, TIC traits, among which the expression of TIC-specific markers, are thought to be reversible. Flow cytometry experiments coupled to Markov model predictions have highlighted that different purified breast cancer cell populations display extensive plasticity and always return to a phenotypic proportion equilibrium over time [151]. In addition, epigenetic changes could directly influence marker expression [49]. Future studies that systematically address the expression of TIC markers, combined with genomic and transcriptomic profile analysis of single cells will help elucidating the controversies regarding TIC markers. Importantly, recent evidence shows that TICs are highly influenced by the tumor microenvironment [28]. Stromal cell-secreted factors, such as Wnt cascade modulators and TGF-β signals have been shown to restore the TIC phenotype in more differentiated tumor cells [111], thereby increasing TIC frequency [10], both in vitro and in vivo. These observations are in line with the dynamic model, which suggests that TIC features might get restored in a subset of cells after specific TIC eradication, contributing to disease relapse when therapy is arrested [112]. A better understanding of how TICs interact with their microenvironment will thus be crucial for the successful development of TIC-specific therapies.

## Conclusion

Enormous progresses have been made over the last years in TIC research. However, comprehensive understanding on how to specifically isolate and target the aggressive subset of TICs still needs to improve. Many studies have supported spheroid cultures to be an appropriate mean to enrich for a cell population that displays TIC characteristics. Notwithstanding, xenotransplantation of cells performed in limiting dilution conditions and subsequent tumor formation after serial transplantation in multiple secondary recipients is considered the gold standard in TIC research. Recent evidence illustrates patient-derived spheroid cultures to be a better model to test for CRC therapies than adherent counterparts, not because of enhanced self-renewal potential, but principally because these cultures successfully maintain their resistance to chemotherapeutics. Importantly, several recent studies have unraveled a high plasticity of TICs, a phenomenon that needs to be closely examined for the development of TIC-targeted therapies. Additionally, the metabolic identity of TICs is an emerging field of research and targeting TIC metabolism seems to represent a promising approach for the development of new TIC-specific treatments. To conclude, the development of strategies that exploit the unique characteristics of TICs, without neglecting the impact of inter- and intra-tumor heterogeneity, will hopefully result in the specific eradication of TICs, thereby eventually preventing disease progression and recurrence. Lastly, it is important to mention that the CSC and clonal evolution concepts are not, as initially suggested, mutually exclusive and thus efficient therapies will include targeting both populations, the fast-diving tumor cells as well as TICs.

## Abbreviations

5-FU: 5-fluorouracil
ABC: ATP-binding cassette
ABCG2: ATP binding cassette subfamily G member 2
ALDH1: Aldehyde dehydrogenase 1 (ALDH1)
AML: Acute myeloid leukemia
APC: Adenomatous polyposis coli
BMI-1: B lymphoma Mo-MLV insertion region 1 homolog polycomb ring finger
BMP4: Bone morphogenetic protein 4
BrdU: Bromodeoxyuridine
CRC: Colorectal cancer
CSCs: Cancer stem cells
DLL4: Delta-like canonical Notch ligand 4
EMT: Epithelial to mesenchymal transition
EpCAM/ESA: Epithelial-specific antigen
HIF-1α: Hypoxia-inducible factor 1α
ID: Inhibitor of DNA binding
IL-4: Interleukin 4
iNOS: Inducible nitric oxide synthase
JAK: Janus kinase
PDH: Pyruvate dehydrogenase
PDK1: Pyruvate dehydrogenase kinase 1
PRC1: Polycomb regulatory complex 1 (PRC1)
SC: Spheroid culture
SP: Side population
STAT3: Signal Transducer and Activator of Transcription 3
TGF-β: transforming growth factor-β
TIC: Tumor-initiating cell
